# Application of non-invasive prenatal testing to 91,280 spontaneous pregnancies and 3477 pregnancies conceived by in vitro fertilization

**DOI:** 10.1186/s13039-023-00656-y

**Published:** 2023-09-19

**Authors:** Rong Wei, Jingran Li, Yuanyuan Xia, Chaohong Wang, Xinran Lu, Yuqin Fang, Jiansheng Zhu

**Affiliations:** 1 Affiliated Maternity and Child Health Hospital of Anhui Medical University, Hefei, China; 2 The Fifth Clinical Medical College of Anhui Medical University, Hefei, China

**Keywords:** Non-invasive prenatal testing (NIPT), Assisted reproductive technology (ART), Cell-free DNA, In vitro fertilization (IVF), Sex chromosome aneuploidy (SCA), Chromosomal aneuploidies, Next-generation sequencing (NGS)

## Abstract

**Background:**

Many clinical studies based on spontaneous pregnancies (SPs) have demonstrated the superiority of non-invasive prenatal testing (NIPT), and the question of whether this technology is suitable for offspring conceived by assisted reproductive technology has attracted attention. This study aimed to evaluate the application value of NIPT in screening for trisomy (T)21, T18, T13 and sex chromosome aneuploidy (SCA) in pregnant women who conceived by in vitro fertilization (IVF).

**Results:**

In total, there were 804 high-risk cases [0.88% (804/91280), singleton = 795, twin = 9] in the SP group. Among the 558 invasive prenatal diagnosis (IPD) cases (singleton = 556, twin = 2), 343 (singleton = 342, twin = 1) were true positive, including 213 cases of T21, 28 of T18, 5 of T13 and 97 (singleton = 96, twin = 1) of SCA. The positive predictive values (PPVs) of T21, T18, T13, SCA and T21/T18/T13 combined in singleton pregnancy were 89.12% (213/239), 51.85% (28/54), 21.74% (5/23), 40.00% (96/240), and 77.85% (246/316), respectively, and the PPV of SCA in twin pregnancy was 100.00%. In the IVF group, IPD was performed in 19 (singleton = 16, twin = 3) of the 27 high-risk cases [0.78% (27/3477), singleton = 16, twin = 3], of which 9 (singleton = 8, twin = 1) were true positive, including 5 cases (singleton = 4, twin = 1) of T21 and 4 of SCA. The PPVs of singleton T21, SCA and T21/T18/T13 combined were 66.67% (4/6), 50.00% (4/8) and 57.14% (4/7), respectively, and the PPV of twin T21 was 100.00% (1/1). There were no significant differences in PPV among T21, SCA and T21/T18/T13 combined in singletons between the groups (89.12% vs. 66.67%, *p* = 0.09; 40.00% vs. 50.00%, *p* = 0.57; 77.85% vs. 57.14%, *p* = 0.20). The sensitivity and specificity were higher for singleton and twin pregnancies in the two groups. Based on follow-up results, 1 case of false negative T21 was found in the singleton SP group. Additionally, the mean foetal fraction (FF) of the IVF group was lower than that of the SP group (11.23% vs. 10.51%, *p* < 0.05).

**Conclusion:**

NIPT has high sensitivity and specificity in screening chromosomal aneuploidies in both IVF pregnancy and spontaneous pregnancy, so it is an ideal screening method for IVF pregnancy.

## Background

Currently, infertility is a highly prevalent global disease, and according to the WHO statistics, infertility affects approximately 8–12% of the global population [1]. Studies have shown that the prevalence of infertility in China is approximately 12–18% and is on the rise. Clinically, assisted reproductive technology (ART) is effective for the treatment of infertility, and in vitro fertilization-embryo transfer (IVF-ET) is a widely used method [2]. With the increasing proportion of offspring conceived by ART in the population, there is growing concern about the safety of ART. In particular, China has a high incidence of birth defects, with an estimated total annual incidence of approximately 5.6% and number of new birth defects of approximately 900,000 per year [3]. It has been reported that children conceived by ART have an increased risk of birth defects compared to those conceived by SP [4], and traditional serological screening methods perform poorly in IVF. A significant decrease in serum marker pregnancy-associated plasma protein A (PAPP-A) in pregnant women with ART leads to an increase in the false positive rate (FPR) [5], resulting in an increase in the number of patients undergoing invasive prenatal diagnosis (IPD). Chorionic villus sampling (CVS) and amniocentesis are the gold standard methods for prenatal diagnosis, but they have an approximately 0.1–0.3% risk of abortion [6]. This increases anxiety among women who become pregnant by ART, so compliance with the recommendation to undergo IPD is low among these women. Therefore, among clinicians and pregnant women, there is an urgent need to have a screening method that is both safe and efficient to reduce the incidence of birth defects in IVF pregnancies.

Non-invasive prenatal testing (NIPT) involves the application of molecular genetic techniques such as next-generation sequencing (NGS) to detect foetal cell-free DNA (cfDNA) in maternal plasma during pregnancy to assess the risk of common foetal chromosomal aneuploidies, with the main target diseases including T21, T18 and T13. Due to improvements in this technology, SCAs can also be detected. In 1997, it was first reported that maternal plasma contained foetal cfDNA [7]. Later, it was confirmed that most foetal cfDNA came from placental trophoblast cells, and the concentration of foetal cfDNA increased with gestational age [8, 9]. Fan et al. [10] and Chiu et al. [11] reported the results of large-scale sequencing study based on the whole genome to detect foetal chromosome aneuploidy in peripheral blood, which proved the feasibility of a new method involving NGS to detect foetal chromosome diseases in maternal plasma by analysing cfDNA. Since 2011, NIPT has been rapidly applied in the clinic. NIPT is now performed globally, and many countries have adopted NIPT as part of their clinical screening. Although many studies have demonstrated the advantages of NIPT [12–15], NIPT has limitations. In the Technical Specification for Prenatal Screening and Diagnosis of Foetal Free DNA in Peripheral Blood of Pregnant Women issued by the General Office of the Health and Family Planning Commission in China, pregnant women with IVF-ET pregnancies are classified as a population that requires caution [16]. At present, only a few studies have reported the feasibility of NIPT in ART [17–19], and large-scale population studies are lacking. Therefore, more large-scale clinical data of pregnant women treated by IVF are needed to demonstrate the effectiveness of NIPT in ART.

The objective of this study was to evaluate the feasibility and clinical application of NIPT in screening for T21, T18, T13 and SCA diseases in 3477 pregnancies conceived by IVF by comparing them with the results obtained for 91,280 SPs and to provide a basis for quality assurance of NIPT for large-scale clinical application.

## Materials and methods

### Study subjects

**Fig. 1:**
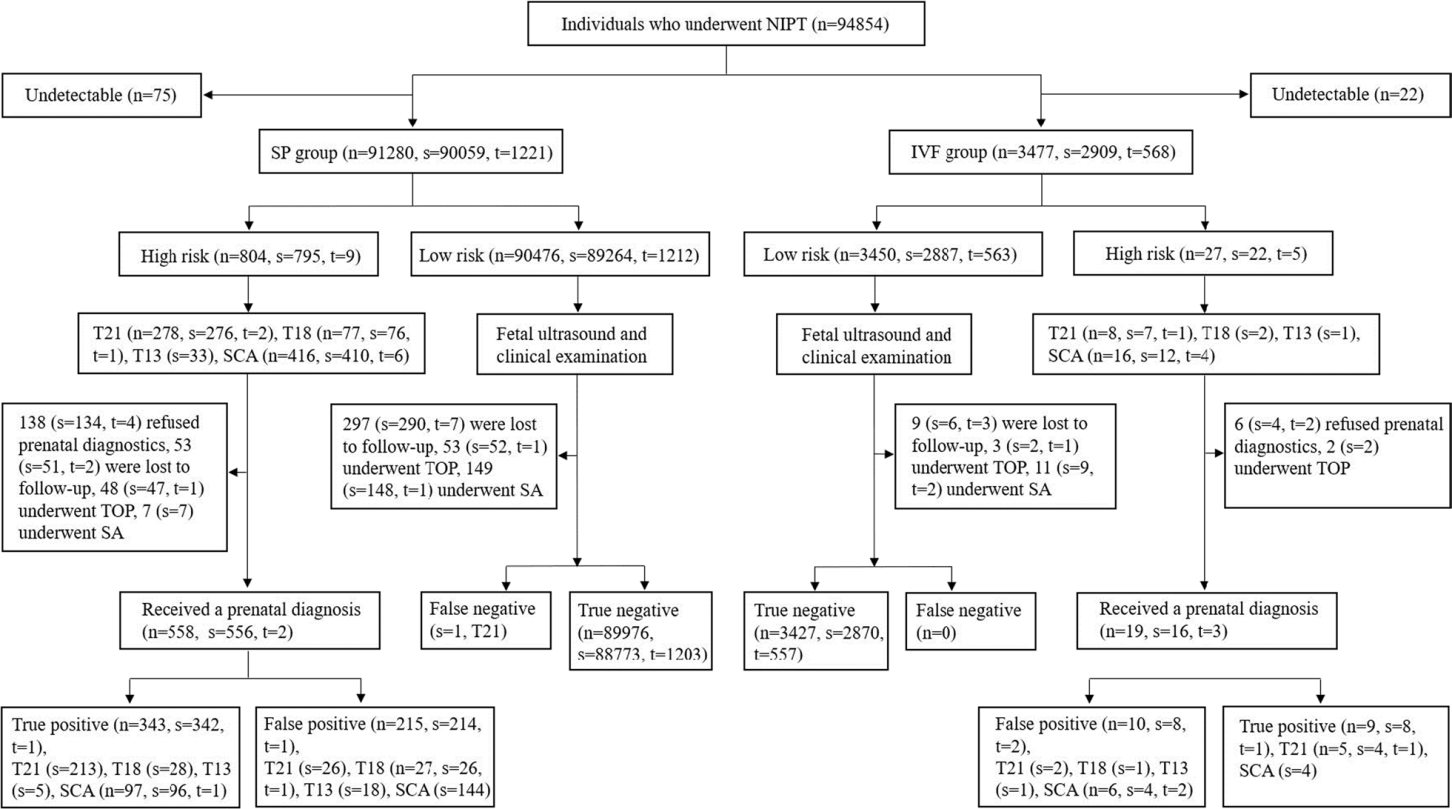
Flow chart of the NIPT study. *NIPT* non-invasive prenatal testing, *T21* trisomy 21, *T18* trisomy 18, *T13* trisomy 13, *SCA* sex chromosome aneuploidy, *SP* spontaneous pregnancy, *IVF* in vitro fertilization, *n* number, *s* singleton, *t* twin, *TOP* termination of pregnancy, *SA* spontaneous abortion

A total of 94,757 pregnant women who received NIPT at Anhui Maternal and Child Health Hospital from January 2016 to June 2022 were selected. In our study, we divided pregnant women into two groups according to the mode of conception, namely, the SP group and the IVF group. The SP group included 91,280 pregnant women, of whom 90,059 (98.66%, 90,059/91,280) had singleton pregnancies and 1221 (1.34%, 1221/91280) had twin pregnancies. The mean age, BMI, and gestational age of the pregnant women were 30.16 years (range 18–48 years; SD: 4.60), 23.52 kg/m^2^ (range 14.01–57.10 kg/m^2^; SD: 3.73), and 16^+4^ weeks (range 12^+0^–26^+6^ weeks), respectively. In the IVF group of 3477 pregnant women, there were 2909 (83.66%, 2909/3477) cases of singleton pregnancy and 568 (16.34%, 568/3477) cases of twin pregnancy, the mean age, BMI, and gestational age of the pregnant women were 31.23 years (range 21–48 years; SD: 4.23), 24.14 kg/m^2^ (range 21.32–37.50 kg/m^2^; SD: 3.55), and 16^+5^ weeks (range 12^+0^–25^+4^ weeks) (Table 1 and Fig. 1).

**Table 1:** Demographic and pregnancy characteristics of NIPT-screened pregnant women

Characteristic	SP group	IVF group	*p* value
Maternal age			
Age range (years)	18–48	21–48	–
Older parturient women (≥ 35 years, n, %)	16,977 (18.60%)	793 (22.81%)	< 0.05
≤ 29 years (n, %)	41,154 (45.09%)	1234 (35.49%)	< 0.05
30–34 years (n, %)	33,149 (36.32%)	1450 (41.70%)	< 0.05
35–39 years (n, %)	15,692 (17.19%)	640 (18.41%)	0.06
≥ 40 years (n, %)	1285 (1.41%)	153 (4.40%))	< 0.05
Average age (SD, years)	30.16 (4.60)	31.23 (4.23)	< 0.05
Gestational age			
Gestational age range (weeks)	12^+0^–26^+6^	12^+0^–25^+4^	–
12^+0^–15^+6^ weeks (n, %)	12,204 (13.37%)	1337 (38.45%)	< 0.05
16^+0^–19^+6^ weeks (n, %)	75,103 (82.28%)	2026 (58.27%)	< 0.05
20^+0^–23^+6^ weeks (n, %)	3014 (3.30%)	94 (2.70%)	0.05
24^+0^–26^+6^ weeks (n, %)	959 (1.05%)	20 (0.58%)	< 0.05
Mean gestational age (weeks)	16^+4^	16^+5^	–
Type of pregnancy			
Singleton (n, %)	90,059 (98.66%)	2909 (83.66%)	< 0.05
Twin (n, %)	1221 (1.34%)	568 (16.34%)	< 0.05
Body mass index (BMI, kg/m^2^)			
BMI range (kg/m^2^)	14.01–57.10	21.32–37.50	–
BMI average value (SD, kg/m^2^)	23.52 (3.73)	24.14 (3.55)	0.48
Total (n)	91,280	3477	–

*SP* spontaneous pregnancy, *IVF* in vitro fertilization, *BMI* body mass index, *SD* standard deviation; –, no statistical analysis or calculation failure, *p* probability

### Indications for NIPT

The inclusion criteria were as follows: (1) pregnant women with IVF-ET pregnancies; (2) pregnant women with gestational weeks and age range of 12^+0^–26^+6^ weeks and 18–48 years; (3) pregnant women with high (T21 risk value ≥ 1/270, T18 risk value ≥ 1/350) and critical risk (T21 risk value = 1/270–1/1000, T18 risk value = 1/350–1/1000); and (4) pregnant women who missed Down's syndrome screening and volunteered for NIPT screening. All pregnant women who underwent NIPT screening received a pre-test genetic counselling workup and signed written informed consent before blood collection.

The exclusion criteria were as follows: (1) gestational age and age range beyond the range of 12^+0^–26^+6^ weeks and 18–48 years, respectively; (2) a clear chromosome abnormality in either the husband or wife; (3) transplantation, stem cell treatment, or allogeneic blood transfusion received by one member of the couple within one year or cellular immunotherapy with exogenous DNA within 4 weeks; (4) foetal ultrasound examination showing structural abnormalities; (5) a family history of genetic disease or suggestive of a high risk of genetic disease; or (6) pregnancy complicated by malignancy.

In this study, pregnant women who met the criteria of the applicable population and underwent the test or volunteered to undergo the test were informed in detail about the target disease, purpose, significance, accuracy, limitations, risks and other screening and diagnostic options to the pregnant women and their families, and the pregnant women or their families signed the informed consent form and completed in the application form.

### Non-invasive prenatal testing (NIPT)

The peripheral blood (5 ml) of pregnant women was collected in cell-free DNA BCT blood collection tubes. Immediately after collection, it was gently inverted up and down 8–10 times to mix thoroughly, and the samples were uniquely numbered. Plasma was separated by a double centrifugation procedure (whole blood centrifuged at 1600×*g* for 10 min at 4 °C, supernatant aspirated; supernatant centrifuged at 16,000×*g* for 10 min at 4 °C), and 0.6 ml of supernatant plasma was aspirated into 2.0 ml centrifuge tubes stored at − 80 °C in a refrigerator to avoid repeated freeze‒thawing. The cfDNA in the samples was extracted using the Plasma cfDNA Extraction Kit (Berry Genomics, China), and the eluted DNA was quantified using a Qubit® 2.0 Fluorometer (Life Technology). The high-throughput sequencing library construction DNA purification kit (Berry Genomics, China) was used for library construction (DNA end repair, connecting sequencing adapter, library purification) and library quantification, and high-throughput sequencing was performed by an Illumination NextSeq CN500 sequencer. The sequencing results were transferred to the data analysis system (V1.0, Berry Genomics, China) for sequence alignment and statistical analysis to obtain Z scores. Foetal aneuploidy status for whole chromosomes was determined by Z scores (Z ≥ 3.0 or Z ≤ − 3.0, high risk; − 3.0 < Z < 3.0, low risk). The foetal fraction (FF) is the proportion of foetal cfDNA in maternal blood. In this study, FF was calculated in two parts: the male foetal fraction was estimated based on the content of the Y chromosome, while the female foetal fraction was estimated based on the fragment size distribution of cfDNA. The detection threshold of the foetal fraction was set at 4%, and only at FF ≥ 4% could the Z score be calculated. If FF was below the threshold, retesting or blood sampling was required [20, 21]. It was recommended that all pregnant women with high-risk results of NIPT receive professional genetic counselling and undergo IPD to verify the NIPT results.

### Invasive prenatal diagnosis (IPD)

IPD was performed after genetic counselling was provided to and informed consent obtained from the pregnant women for which NIPT findings suggested a high risk. Amniocentesis was performed using ultrasound, and cell culture amniotic fluid karyotyping and chromosome microarray analysis (CMA) were performed according to prenatal diagnostic procedures.

### Karyotype analysis

Chromosome preparation was performed by the amniotic fluid in situ method through standardized cell culture operating procedures. Amniotic fluid samples (20 ml) were collected in sterile tubes and centrifuged at 1500×*g* for 5 min at room temperature. The supernatant was discarded, and the samples were inoculated in amniotic fluid medium and incubated in a 37 °C incubator with 5% CO_2_. By the 7th day of culture, 8–10 patches of growing amniotic fluid cell colonies were observed under an inverted microscope for chromosome analysis, and for late pregnancy samples, the culture time was extended if the number of cell colonies was insufficient. After obtaining the amniotic fluid cell set, changing the solution and continuing to culture for 5 h, we added colchicine overnight to stop cell division at the mid-division stage, obtained chromosome specimen films after hypotonicity, fixation, filming, G banding and staining procedures, and performed karyotype analysis according to the standard ISCN (2016).

### Chromosome microarray analysis (CMA)

Amniotic fluid (10 ml) was centrifuged at 1600×*g* for 10 min at 4 °C, the supernatant was discarded to retain precipitation, and samples for chromosomal microarray analysis were extracted using the CytoScan kit (Affymetrix, USA) in strict accordance with the laboratory standard operating procedure and the instructions of the kit. Affymetrix CytoScan 750 K SNP-Array (Affymetrix, USA) was used for genome-wide scanning assays, and the results were analysed by chromosome analysis suite software. The results were analysed with reference to public databases (CLINGEN, DECIPHER, CLINV AR, OMIM, DGV, ISCA, NCBI, UCSC) and the American College of Medical Genetics (ACMG) guidelines [22].

### Statistical methods

SPSS 25.0 software was used to analyse the results. The data are expressed as the number and percentage of cases (%). Sensitivity, specificity, positive predictive value, negative predictive value, positive rate and false positive rate were calculated. The t test, χ^2^ test or Fisher's exact test was used to compare the relevant results between the two groups, and *p* < 0.05 was considered statistically significant.

### Follow-up of pregnancy outcome

All participants were followed up to evaluate pregnancy outcomes 1 month after the expected date of delivery through inpatient information queries and telephone follow-up. Follow-up information included data on delivery outcomes (delivery, labour induction, or miscarriage), physical examination of the new-born, and the presence or absence of birth defects (physical examination or genetic diagnosis).

## Results

### Overall screening results of NIPT samples

A total of 94,854 NIPT samples were tested. Among them 94,757 were tested successfully, and 97 (SP = 75, IVF = 22) were not tested successfully. The overall failure rate was 0.10% (97/94,854), and the failure rates of the SP group and IVF group were 0.08% (75/91,355) and 0.63% (22/3499), respectively. The proportions of older pregnant women (maternal age ≥ 35 years) in the SP group and the IVF group were 18.60% (16,977/91,280) and 22.81% (793/3477), respectively, and the proportions of twin pregnancies were 1.34% (1221/91,280) and 16.34% (568/3477), respectively (Fig. 1 and Table 1).

In the SP group, there were 91,280 samples (singleton = 90,059, twin = 1221). Among them, NIPT revealed 804 high-risk cases (0.88% (804/91280), singleton = 795, twin = 9), of which there were 278 cases (singleton = 276, twin = 2) of T21, 77 (singleton = 76, twin = 1) of T18, 33 of T13 and 416 (singleton = 410, twin = 6) of SCA. IPD was performed in 558 cases (singleton = 556, twin = 2), of which 343 cases (singleton = 342, twin = 1) were true positive and 215 cases (singleton = 214, twin = 1) were false positive. In the IVF group of 3477 samples (singleton = 2909, twin = 568), there were 27 high-risk cases [0.78% (27/3477), singleton = 22, twin = 5], including 8 cases (singleton = 7, twin = 1) of T21, 2 cases of T18, 1 case of T13 and 16 cases (singleton = 12, twin = 4) of SCA. IPD was performed in 19 cases (singleton = 16, twin = 3), of which 9 cases (singleton = 8, twin = 1) were true positive and 10 cases (singleton = 8, twin = 2) were false positive (Fig. 1  and Table 2).

Data on the pregnancy outcomes of the remaining NIPT-positive cases without a prenatal diagnosis were obtained through neonatal physical examination after delivery and from the records of adverse pregnancy outcomes. A total of 144 patients (SP = 138, singleton = 134, twin = 4; IVF = 6, singleton = 4, twin = 2) refused to receive a prenatal diagnosis, but the pregnancy outcomes of the new-borns were not obviously abnormal in the clinical phenotypic confirmation. In 53 cases (SP = 53, singleton = 51, twin = 2), there were no data on pregnancy outcomes owing to loss of follow-up. Fifty women (SP = 48, singleton = 47, twin = 1; IVF = 2, singleton = 2) chose termination of pregnancy due to abnormal ultrasound findings or other reasons, and 7 (SP = 7, singleton = 7) had a spontaneous abortion (SA) but no confirmatory genetic testing results for the products of conception (Fig. 1  and Table 2).

All pregnant women with low-risk NIPT were followed up for pregnancy outcomes. There were 93,403 cases (SP = 89,976, singleton = 88,773, twin = 1203; IVF = 3427, singleton = 2870, twin = 557) of live birth with normal phenotypic confirmation results. There were no data on pregnancy outcomes in 306 cases (SP = 297, singleton = 290, twin = 7; IVF = 9, singleton = 6, twin = 3) owing to loss of follow-up. A total of 160 women (SP = 149, singleton = 148, twin = 1; IVF = 11, singleton = 9, twin = 2) had a SA, and 56 (SP = 53, singleton = 52, twin = 1; IVF = 3, singleton = 2, twin = 1) chose termination of pregnancy due to abnormal ultrasound findings or other reasons but had no confirmatory genetic testing results for the products of conception. In addition, there was 1 false negative T21 in the SP group, which was confirmed by cytogenetics (Fig. 1  and Table 2).

Therefore, in this population of 93,981 (SP = 90,535, singleton = 89,330, twin = 1205; IVF = 3446, singleton = 2886, twin = 560) pregnancies, cytogenetic or phenotypic confirmation of NIPT results was available in 577 (SP = 558, singleton = 556, twin = 2; IVF = 19, singleton = 16, twin = 3) NIPT high-risk cases and 93,404 (SP = 89,977, singleton = 88,774, twin = 1203; IVF = 3427, singleton = 2870, twin = 557) NIPT low-risk cases. Further calculation of NIPT sensitivity and specificity was based on this subgroup of the population with outcome data available.

### The effectiveness of NIPT for T21/T18/T13 and SCA

In the SP group, a total of 558 pregnant women underwent IPD after obtaining informed consent, with a prenatal diagnosis rate of 69.40% (558/804). Among them, 343 were true positives, including 213 cases of T21, 28 cases of T18, 5 cases (singleton = 5) of T13 and 97 cases (singleton = 96, twin = 1) of SCA. The positive predictive values (PPVs) of singleton T21, T18, T13 and SCA were 89.12% (213/239), 51.85% (28/54), 21.74% (5/23) and 40.00% (96/240), respectively; the sensitivity and specificity were 99.53% and 99.97%, respectively, for T21, 100.00% and 99.97%, respectively, for T18, and 100.00% and 99.98%, respectively for T13, and the combined PPV for T21/T18/T13 was 77.85% (246/316). The PPV of twin SCA was 100.00% (1/1), and the specificity values for T21, T18 and T13 were 100.00%, 99.92% and 100.00%, respectively. In the IVF group, 19 cases were verified for IPD, with a prenatal diagnosis rate of 70.37% (19/27), of which 9 cases were true positives, including 5 cases (singleton = 4, twin = 1) of T21 and 4 cases (singleton = 4) of SCA, with PPVs of 66.67% (4/6) and 50.00% (4/8) for T21 and SCA, respectively in singletons. The sensitivity and specificity of T21 were 100.00% and 99.93%, respectively, the specificity of T18 and T13 was 99.97% and 100.00%, respectively, and the combined PPV of T21/T18/T13 was 57.14% (4/7). The PPV of twin T21 was 100.00% (1/1), and the sensitivity and specificity of T21 and the specificity of T18 and T13 were all 100.00% (Tables 2 and 3). The details of prenatal diagnosis results and pregnancy outcomes of 27 high-risk NIPT cases in the IVF group are shown in Table 4.

**Table 2:** Effectiveness of NIPT screening for T21/T18/T13/SCA

Characteristic	SP group								IVF group							
	Singleton				Twin				Singleton				Twin			
NIPT result	T21	T18	T13	SCA	T21	T18	T13	SCA	T21	T18	T13	SCA	T21	T18	T13	SCA
High-risk result (n)	276	76	33	410	2	1	0	6	7	2	1	12	1	0	0	4
Diagnostic results (n)	239	54	23	240	0	1	0	1	6	1	0	8	1	0	0	2
TP (n)	213	28	5	96	0	0	0	1	4	0	0	4	1	0	0	0
FP (n)	26	26	18	144	0	1	0	0	2	1	0	4	0	0	0	2
TN (n)	89,090	89,276	89,307	–	1205	1204	1205	–	2880	2885	2886	–	559	560	560	–
FN (n)	1	0	0	0	0	0	0	0	0	0	0	0	0	0	0	0
Sensitivity (%)	99.53	100.00	100.00	–	–	–	–	–	100.00	–	–	–	100.00	–	–	–
Specificity (%)	99.97	99.97	99.98	–	100.00	99.92	100.00	–	99.93	99.97	100.00	–	100.00	100.00	100.00	–
PPV (%)	89.12	51.85	21.74	40.00	–	0.00	–	100.00	66.67	0.00	–	50.00	100.00	–	–	0.00
NPV (%)	99.99	100.00	100.00	100.00	100.00	100.00	100.00	100.00	100.00	100.00	100.00	100.00	100.00	100.00	100.00	100.00

*NIPT* non-invasive prenatal testing, *SP* spontaneous pregnancy, *IVF* in vitro fertilization, *TP* true positive, *FP* false positive, *FN* false negative, *TN* true negative, *PPV* positive predictive value, *NPV* negative predictive value; –, no statistical analysis or calculation failure

**Table 3:** Comparison of PPV of T21/T18/T13/SCA between the SP and IVF groups

NIPT result	Singleton		*p* value	Twin		*p* value
	SP group (PPV, %)	IVF group (PPV, %)		SP group (PPV, %)	IVF group (PPV, %)	
T21	89.12	66.67	0.09	–	100.00	–
T18	51.85	0.00	–	0.00	–	–
T13	21.74	–	–	–	–	–
T21/T18/T13	77.85	57.14	0.20	0.00	100.00	–
SCA	40.00	50.00	0.57	100.00	0.00	–

*PPV* positive predictive value, –, no statistical analysis or calculation failure

**Table 4:** Details of prenatal diagnosis results and pregnancy outcomes of 27 high-risk NIPT cases in the IVF group

Patients	Maternal age (years)	Gestational age (weeks)	Pregnancy type	NIPT result	Prenatal diagnosis result	Clinical outcome
Case 1	37	16^+5^	Singleton	T13	46, XN	Delivery
Case 2	40	16^+6^	Singleton	T18	Abnormal ultrasound	Induced labour
Case 3	32	19^+6^	Singleton	T18	46, XN	Delivery
Case 4	34	19^+3^	Singleton	T21	47, XN + 21	Induced labour
Case 5	33	17^+2^	Singleton	T21	47, XN + 21	Induced labour
Case 6	38	17^+2^	Singleton	T21	47, XN + 21	Induced labour
Case 7	28	18^+5^	Twin	T21	47, XN + 21	Induced labour
Case 8	30	12^+3^	Singleton	T21	47, XN + 21	Induced labour
Case 9	32	15^+5^	Singleton	T21	46, XN	Delivery
Case 10	29	17^+3^	Singleton	T21	46, XN	Delivery
Case 11	43	15^+5^	Singleton	T21	Refuse prenatal diagnosis	Premature delivery
Case 12	32	15^+6^	Singleton	45, X	45, X	Induced labour
Case 13	36	17^+1^	Singleton	45, X	46, XN	Delivery
Case 14	32	16^+5^	Twin	45, X	46, XN	Delivery
Case 15	30	16^+5^	Singleton	45, X	46, XN	Delivery
Case 16	31	16^+5^	Singleton	45, X	Refuse prenatal diagnosis	Delivery
Case 17	31	20^+0^	Singleton	45, X	Refuse prenatal diagnosis	Delivery
Case 18	31	18^+0^	Singleton	47, XXX	46, XN	Delivery
Case 19	30	17^+4^	Singleton	47, XXX	46, [] [].14 ps+	Delivery
Case 20	24	17^+3^	Singleton	47, XXX	Refuse prenatal diagnosis	Loss to follow-up
Case 21	32	13^+1^	Singleton	47, XXX	Refuse prenatal diagnosis	Loss to follow-up
Case 22	27	14^+3^	Twin	47, XXX	Refuse prenatal diagnosis	Loss to follow-up
Case 23	30	18^+1^	Singleton	47, XXY	47, XXY	Induced labour
Case 24	39	13^+0^	Singleton	47, XXY	47, XXY	Delivery
Case 25	28	14^+3^	Singleton	47, XXY	47, XXY	Delivery
Case 26	28	16^+3^	Twin	47, XXY	46, XN	Delivery
Case 27	29	17^+1^	Twin	47, XYY	Refuse prenatal diagnosis	Delivery

### Comparison of the correlation between FF and week of gestation in the two groups

**Fig. 2:**
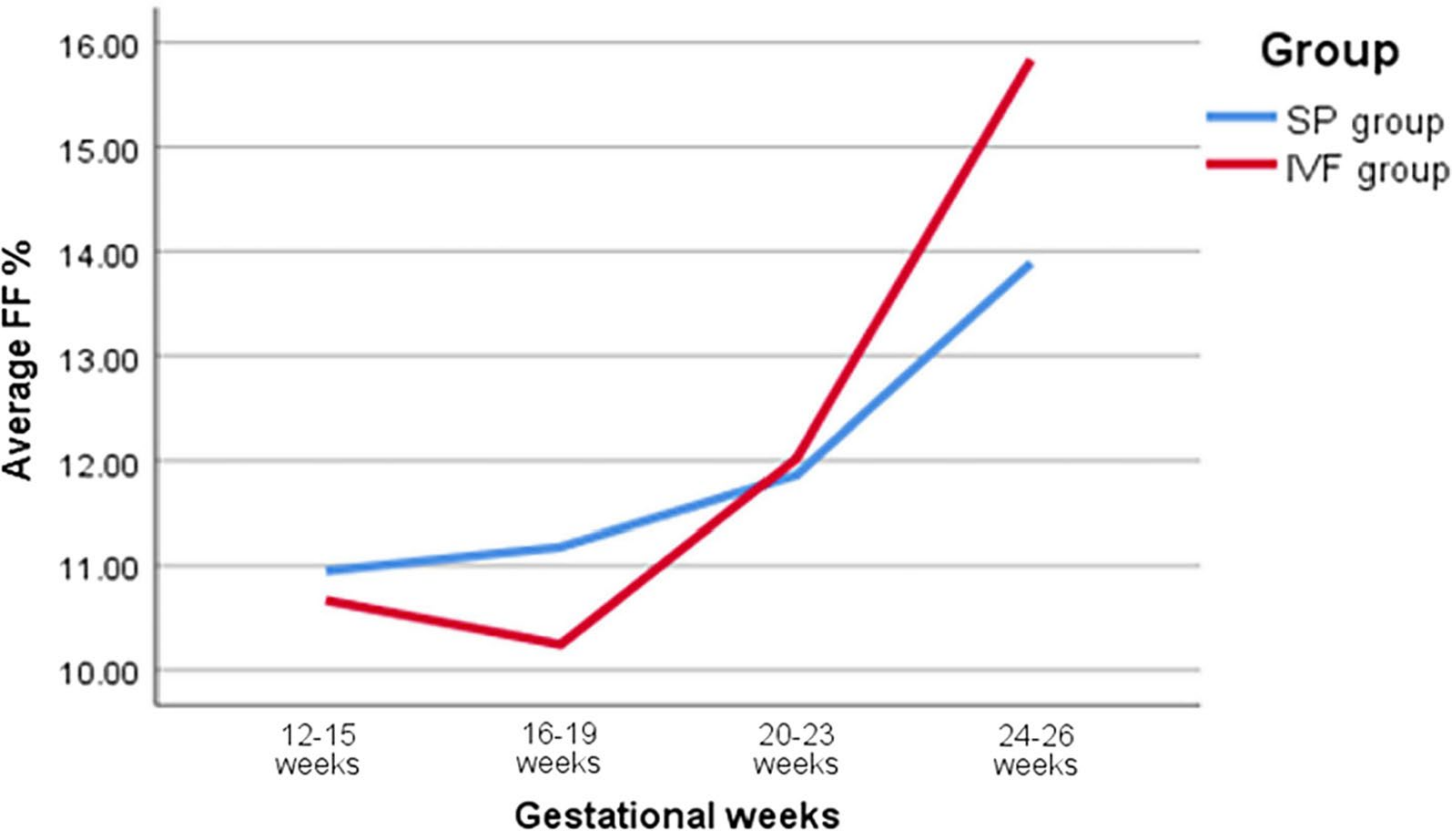
Correlation between gestational age and FF. *SP group* spontaneous pregnancy group, *IVF group* in vitro fertilization group, *FF* foetal fraction

In this study, to study the relationship between FF and gestational weeks, pregnant women in the SP and IVF groups were matched for age, BMI and gestational weeks. Our results showed that the mean FF values of the SP group at 12^+0^–15^+6^ weeks, 16^+0^–19^+6^ weeks, 20^+0^–23^+6^ weeks, 24^+0^–26^+6^ weeks and all gestational weeks were 10.95%, 11.18%, 11.86%, 13.89% and 11.23%, respectively, and they were 10.67%, 10.25%, 12.01%, 15.84% and 10.51%, respectively, in the IVF group. With increasing gestational age, the average FF of the SP group showed an overall increasing trend, although the average FF of the IVF group decreased slightly from 16^+0^ to 19^+6^ weeks but showed an overall increasing trend (Fig. 2). As shown in Fig. 2 and Table 5, there was a significant difference in the mean FF between the two groups at gestational age 16^+0^–19^+6^ weeks (11.18% vs. 10.25%, *p* < 0.05) and the overall gestational age (11.23% vs. 10.51%, *p* < 0.05), with that in the IVF group being lower than that in the SP group.

**Table 5:** Comparison of the correlation between the average FF and gestational age in the two groups

Gestational weeks	SP group		IVF group		t-text	*p* value
	Average FF (%)	SD	Average FF (%)	SD		
12^+0^–15^+6^ (weeks)	10.95	4.14	10.67	4.04	1.26	0.21
16^+0^–19^+6^ (weeks)	11.18	4.18	10.25	4.52	4.55	< 0.05
20^+0^–23^+6^ (weeks)	11.86	4.39	12.01	5.57	− 0.14	0.89
24^+0^–26^+6^ (weeks)	13.89	4.89	15.84	4.46	− 0.97	0.33
Total (weeks)	11.23	4.22	10.51	4.36	4.77	< 0.05

*FF* foetal fraction, *SD* standard deviation

## Discussion

In recent years, NIPT has been proven superior in screening for SP foetal chromosomal aneuploidy disorders by many studies at home and abroad, and it has been recognized as a nearly perfect screening method [12–14]. Galeva Savanna et al. [23] reported that the screening efficacies for twin and singleton pregnancies were similar, especially for T21. This has given hope to the medical community and the public that NIPT can also be applied in ART. However, few studies have reported data on NIPT in ART compared to NIPT in SP. More importantly, existing studies have focused on T21, T18 and T13, with even fewer studies on SCA. Marco La Verde et al. [18] reported that NIPT was performed on 36,456 singleton and twin pregnancies, and the results showed that NIPT had high accuracy and was suitable for both singleton and twin pregnancies, of which the results of the ART group of 1807 cases (403 twin pregnancies) showed that the sensitivity and specificity for T21 and T18 were 100%, and the specificity values for T13 and SCA were 99.94% and 99.83%, respectively. Yang Cuiyu et al. [24] reported that NIPT was performed on 474 twin pregnancies conceived by ART, and the results showed that the PPVs of T21 and T18 were 80.00% (4/5) and 100.00% (1/1), respectively.

The overall NIPT-positive rate was 0.88% in the SP group and 0.78% in the IVF group, with no significant difference between the two groups (0.88% vs. 0.78%, *p* = 0.52), which is consistent with previous studies [12]. In this study, the PPV, sensitivity and specificity of T21 in singleton pregnancy in the SP group were 89.12%, 99.53% and 99.97%, respectively; those in the IVF group were 66.67%, 100.00% and 99.93%, respectively; and the PPV of screening T21 was within 65–94% [25]. In this study, the PPV value of T21 in IVF singleton pregnancy was smaller than that in the SP group, but there was no significant difference between the two groups (66.67% vs. 89.12%, *p* = 0.09) (Table 3). The specificity of twin T21 in the SP group and the PPV, sensitivity and specificity of twin T21 in the IVF group were both 100.00%, consistent with previous studies [12, 14, 17]. Although there was a lack of data on T21 for twins in the SP group and the two groups were not comparable, the PPV of T21 of twins in the IVF group was 100.00%, which may be related to the small sample number of twins. For T18 and T13, the data of the two groups were not comparable due to the lack of data from the IVF group, but their sensitivity and specificity were both ≥ 99.92%, consistent with previous studies [12, 17]. It has been reported that the sensitivity and specificity of NIPT in detecting T21, T18, and T13 are 98.0%-99.6% and 98.8%-99.9%, respectively [14]. In the SP group, the PPV, sensitivity and specificity of singleton T18 were 51.85%, 100.00% and 99.97%, respectively, and those of T13 were 21.74%, 100.00% and 99.98%, respectively. However, the PPV of T13 was lower, which was similar to the results of previous studies [25]. This may account for the low number of T13-positive cases. According to our results, overall, there was no significant difference in the combined PPV of T21/T18/T13 between the SP group and the IVF group in singleton pregnancies (77.85% vs. 57.14%, *p* = 0.20) (Table 3), consistent with previous studies [15, 25, 26]. In conclusion, NIPT has a high detection rate, sensitivity and specificity in screening chromosomal aneuploidies in singleton pregnancies and is an ideal screening method for SP pregnant women and IVF pregnant women. However, large-scale clinical data are needed to demonstrate the effectiveness of NIPT in twin pregnancies in future studies.

In this study, the PPV of singleton SCA was 40.00% in the SP group and 50.00% in the IVF group, and there was no significant difference between the two groups (40.00% vs. 50.00%, *p* = 0.57) (Table 3), consistent with previous studies [26, 27]. Among the twins, the lack of data on SCA in the IVF group led to no comparability between the two groups, but the PPV of SCA in the SP group was 100.00%, which was probably because of the lack of data for the twins. In this study, we did not calculate the sensitivity and specificity of SCA. The reasons for this are as follows: first, the infants with SCA were not found to have obvious phenotypic abnormalities in the clinical physical examination at birth, and most foetuses with SCA were not diagnosed in the neonatal period [28]. Second, the follow-up time of this study was limited, and the follow-up time was up to one month after birth, so the analysis of false negative results of SCA may not be accurate.

In addition, according to our study, the lower PPV of SCA compared with that of autosomal aneuploidies may be due to the lower prenatal diagnosis rate of pregnancies with a high risk of SCA suggested by NIPT than those with a high risk of common trisomy (58.10%, 251/432; 81.45%, 325/399, *p* < 0.05). During follow-up, some pregnant women with NIPT results indicating a high risk of SCA chose to terminate the pregnancy due to abnormal ultrasound findings and other factors during prenatal screening and did not undergo foetal chromosome examination. Because of this, karyotype analysis could not be performed, resulting in the exclusion of these high-risk cases when calculating the PPV of SCA, which may result in a low PPV. In conclusion, NIPT is suitable for SPs and IVF pregnancies when screening for SCA in singleton pregnancies, but more large-scale clinical data are needed to evaluate the effectiveness of NIPT screening for SCA in twin pregnancies. However, for the sake of the health of children conceived by IVF, we recommend that pregnant women with a high risk of SCA by NIPT undergo further prenatal diagnosis to determine the foetal karyotype to reduce the occurrence of sex chromosome birth defects. In addition, it is necessary to extend the follow-up time of children at high risk of SCA to enable the early detection of children with SCAs and implement treatment measures as early as possible.

According to our study, NIPT showed high sensitivity and specificity in SP and IVF. Although NIPT is recognized as an ideal screening method for chromosomal aneuploidy, it cannot avoid the occurrence of false negatives and false positives. In our study, the follow-up results showed that there was only 1 false negative case of T21 in the SP group. The NIPT result indicated a low risk of T21 in this case, and the amniotic fluid karyotype analysis verified that it was mosaicism of 47, XY, + 21 [67]/46, XY [16]. Therefore, it can be seen that the false negative result in this case was caused by the foetal chromosome karyotype of mosaicism, which is consistent with previous studies [15]. Ying et al. [29] reported that mosaicism is an important factor affecting NIPT, that mosaicism of the placenta may reduce the accuracy of the examination, and that mosaicism is the main cause of false negative NIPT findings. In addition, NIPT detection is affected by many factors. Since foetal cfDNA is derived mainly from the trophoblast cells of the placental villi and because the continuous turnover of cytotrophoblasts induces apoptosis to release cfDNA into the maternal blood and does not fully represent the foetus, differences in genetic information between placental and foetal tissue may influence NIPT [7–9]. Yang et al. [30] and Mi et al. [31] reported that the development of ART led to a significant increase in the number of lost twin pregnancies, that residual foetal cfDNA in deceased twins could persist for 16 weeks and that residual foetal cfDNA may affect NIPT outcomes. Tatjana et al. [32] reported that maternal malignancy can also affect NIPT. In conclusion, it is inevitable that NIPT will be affected by multiple factors, although NIPT is a safe and efficient screening method that can effectively help avoid the occurrence of birth defects. However, NIPT is only a prenatal screening method and cannot replace IPD. Therefore, for the health of children, it is recommended that pregnant women with low-risk NIPT findings also receive professional genetic counselling and regular prenatal tests.

The reasons for the failure of the 97 samples included low FF, sample haemolysis, and refusal by the pregnant woman to undergo another blood collection. According to our data analysis, the IVF group had a significantly higher detection failure rate than the SP group (0.63% vs. 0.08%, *p* < 0.05), which is consistent with the results reported by Galeva et al. [23] Their study showed that IVF was the most important factor leading to the failure of the first foetal cfDNA sampling test compared with SP in singleton and twin pregnancies, and IVF resulted in a 3.8-fold increased risk of test failure compared with SP. Scott et al. [9] reported that FF was a key parameter affecting the performance of NIPT, and at FF < 4%, it had a greater impact on the results. However, FF was influenced by many factors. Galeva et al. [23] reported that 23,495 singleton pregnancies and 928 twin pregnancies were screened for foetal trisomy by foetal cfDNA testing, and the results showed that maternal age, weight, gestational age, twins, mode of conception, and placental protein were independent predictors of cfDNA testing failure. The risk of trial failure was higher among twin pregnancies than among singleton pregnancies, mainly because the proportion of twins conceived through IVF was higher. Qiao et al. [21] reported that NIPT was performed on 2817 singleton foetuses (1409 males and 1408 females) and 86 twins, and the results showed that maternal age, BMI, cfDNA concentration, and the number of twins were negatively correlated with FF. In contrast, gestational age was positively associated with FF. In our study, the mean FF at the overall gestational age was lower in the IVF group than in the SP group (10.51% vs. 11.23%, *p* < 0.05) (Table 5), which was consistent with the study by Talbot [33]. According to our data, the proportion of twins (16.34% vs. 1.34%, *p* < 0.05) (Table 1) in the IVF group and the overall average age (31.23 years vs. 30.16 years, *p* < 0.05) (Table 1) of the pregnant women were significantly higher than those in the SP group, and the proportion of twins in the IVF group was approximately 12 times higher than that in the SP group. There was no significant difference in mean maternal BMI between the IVF and SP groups (23.52 kg/m^2^ vs. 24.14 kg/m^2^, *p* = 0.48) (Table 1). In summary, the significantly higher detection failure rate in the IVF group than in the SP group may be explained by the lower overall FF, the older maternal age, the mode of conception and the significantly higher proportion of twins in the IVF group.

## Conclusion

NIPT has high sensitivity and specificity in screening chromosomal aneuploidies in both IVF pregnancy and SP, so it is an ideal screening method for IVF pregnancies. Therefore, with full informed consent and voluntary use, NIPT can be used to screen common chromosomal aneuploidies and SCA diseases. However, high-risk NIPT findings indicate that women with high-risk pregnancies need detailed ultrasound examinations, professional genetic counselling and further prenatal diagnosis in combination with clinical management to improve screening efficiency and reduce the incidence of birth defects among babies conceived by IVF. In addition, the average FF of IVF pregnancies is lower than that of SPs. This may be related to the mode of conception, but the specific mechanism is not fully understood. Therefore, we will need more large-scale clinical data in future studies to verify this hypothesis.

## Data Availability

The data used and analyses in the study can be obtained from the corresponding author upon reasonable request.
